# Personal recovery in forensic institutions as a political process: the significance of frameworks for clinical practice

**DOI:** 10.1192/bjo.2022.556

**Published:** 2022-08-04

**Authors:** Andrew Shepherd, Jenny Shaw

**Affiliations:** Greater Manchester Mental Health NHS Foundation Trust, Edenfield Unit, Prestwich Hospital, Prestwich, UK; and Centre for Mental Health and Safety, University of Manchester, UK

**Keywords:** Recovery, forensic mental health services, rehabilitation, ethics, history of psychiatry

## Abstract

Support of personal recovery has been a stated goal for many mental health services since the early 2000s. Frameworks such as the CHIME-S described in this issue of *BJPsych Open* provide useful tools for the operationalisation of this in clinical practice. It is important, however, that through this act of normalisation we do not lose sight of the radical implications of personal recovery as a personal and political process taking place within a social world.

It has been nearly 30 years since the publication of the paper that set out the most cited definition of personal recovery:

‘ ... a deeply personal, unique process of changing one's attitudes, values, feelings, goals, skills, and/or roles. It is a way of living a satisfying, hopeful, and contributing life even with limitations caused by illness. Recovery involves the development of new meaning and purpose in one's life as one grows beyond the catastrophic effects of mental illness.’^1^

Building on previous political (relating to power interactions between the individual and the state) and clinical assumptions, this definition set an initial framework for much of the work that has followed in this field – principally led by Shepherd, Boardman and Slade.^2^ Leamy et al^3^ operationalised and defined a conceptual framework of recovery that has acted as a benchmark for the exploration that followed in various institutions and fields of practice. In this issue of *BJPsych Open*, Senneseth et al^4^ continue in this tradition by exploring the workability of the CHIME framework defined by Leamy et al within forensic mental health settings. Their review neatly and clearly explores and summarises the pathways and barriers in relation to each of the five core recovery concepts of CHIME – connectedness, hope, identity, meaning and empowerment – experienced by patients accessing forensic mental healthcare. They further add to this framework through the introduction of the concept of safety and security – using the abbreviation CHIME-S to illustrate the particularity of the forensic environment.

As Senneseth et al note, each of these six concepts takes on a particular resonance when viewed through the prism of forensic mental health practice: the concept of safety and security, for example, presents a particular tension. On the one hand, safety is a prerequisite for all human flourishing^5^ that takes on a particular meaning when considering the experiences of trauma and violence that are ubiquitous among forensic populations. Security then represents a necessary extension and requirement for safety but can also become trapping, separating the individual from others (disconnection), reducing hope, stigmatising identity, reducing access to meaningful activity and experience, and disempowering (cf. the pains of imprisonment described by Sykes^6^ and revisited by Crewe^7^). Physical, procedural and relational security may be seen as necessary and proportionate but they risk becoming antithetical to the concept of recovery-focused care in forensic settings.

## Frameworks are necessary…

Frameworks such as those set out by Senneseth et al provide us with important points of coherence about which our practice can coalesce. This is an essential requirement in the provision of care – particularly when the concepts outlined in these frameworks centralise on ideas such as identity, which can be seen as far removed from arguments in relation to the reduction of symptom burden in the ‘treatment’ of mental disorder. Concepts such as empowerment can be radical and challenging, particularly in forensic settings, where much of the wider discourse focuses on the fear of a violent offender who is often portrayed as overwhelming and overpowering, certainly not a figure in need of empowerment. Yet, the reality remains that most forensic patients are radically disempowered and marginalised because of traumatic and other life experiences. Violence in this context may be another symptom of distress that practitioners are called on to work with the individual to understand (identity work, meaning-making) while never seeking to excuse. The experience of this work can be potentially harmful for practitioners and patients both.^8^ It is also important to note – in this reflection – that recovery as a process is undergone by patients and supported by practitioners: this again speaks to the importance of agency and is a helpful point to hold in mind.

## … but are they sufficient?

The question must be raised, though, as to whether these frameworks are sufficient in and of themselves? Do they provide a sufficient point of coherence and shared understanding to promote change – or are they too radical and antithetical to traditional modes of practice to be adopted? Frameworks such as CHIME and CHIME-S provide mid-range theories that link our underlying ethic of practice to day-to-day clinical considerations. In this sense they are of great value – calling overdue focus on many important matters for consideration in appropriately recovery-focused care settings. However, they also demand critical reflection as political documents for the claims that they make in relation to our practice and the experience of mental disorder and violence.

We would argue that these frameworks provide powerful tools and means of elaboration when exploring care and practice but that their utility in this regard also means that they are removed from the essential nature of the experience from which they emerged. The definition provided by Anthony^1^ represents a synthesis of preceding discourse – emerging from the sphere of disability rights, as well as feminist and survivor movements.^9^ In these arguments the ‘personal’ in personal recovery is firmly equated with the political, and powerful survivor voices emerge, providing ideographic accounts of their experience,^10,11^ displaying meaning-making that challenges the central notion of Anthony's definition ‘ … with limitations caused by illness … ’ by questioning the way mental disorder is considered. Viewed in this light, and historical context, these frameworks are political documents that demand a renewed focus on individual power and experience. This becomes particularly challenging in forensic settings, where a collective desire to look away from trauma and violence can become overwhelming.^12^

## Critical evaluation: a role for psychological jurisprudence

Tools are therefore needed that allow us to consider the claims made by these frameworks through a critical lens. Psychological jurisprudence^13^ provides us with one such set of tools (among others – see for example Mullen)^14^ and we offer a brief exploration here. Psychological jurisprudence sets out three primary assumptions: (a) the unconscious exists and is structured like a language; (b) subjectivity is always politicised; and (c) power functions through politics and language. Through this lens the ‘mentally disordered offender’, as the object of forensic mental health practice, can be seen as an individual who is simultaneously feared and rejected by society. Incarceration and detention, as responses to violence, ostensibly seek to disempower; but removing the individual from scrutiny also allows them to become the object of fantastic imagination and projection – taking on the form of a threatening and terrifying figure. Language emerges through a corpus such as the Mental Health Act and criminal law that seeks to codify and contain such a figure, while the public imaginary continues its own work apace. Through the technologies of forensic mental health practice (diagnostic formulation, risk assessment, security, treatment) we enact and embody the flow of power in this discourse. As carers, professionals and actors in this field we too are subject to projections both from wider society, our patients and our peers.^8^ Disrupted attachment dynamics, institutional anxiety and fear of violence all contribute through well-recognised defence processes (such as splitting) to undermine our capacity for thought – risking the emergence of more primitive responses, such as a sadistic urge to punish, that are antithetical to the concepts of recovery and good clinical care. Frameworks such as CHIME-S can, in this light, be seen as reductive, as an effort to codify and map human suffering and distress through six-letter abbreviations that are far removed from the powerful political and personal narratives of the psychiatric survivor movement. However, from another perspective they serve to remind us of an essential truth that runs true between recovery as described by the survivor movement and as operationalised from the definition given by Anthony: recovery is a process of work, or emotional labour, that occurs within a social space (connectedness) and is essentially political in its nature (identity, meaning and empowerment). The requirement for safety and security in this light can be seen as a human requirement for the development of identity, hope and personal flourishing.^5,15,16^

## Conclusions

In closing then, frameworks such as the CHIME-S are powerful clinical tools that are in many ways still radical in relation to forensic clinical practice. They are also, however, powerful political statements that draw on a long and contested history in their development. If misapplied, as an end in themselves, they have the potential to further reduce and marginalise the experience of mentally disordered offenders through a segmentation of their experience and subjectivity into neat dividing concepts, disavowing the personal experience and narrative of the individual within a social context that has led to their current situation. For example, in a recent exploration of a structured professional judgement measure (DUNDRUM – the Dangerousness, Understanding, Recovery and Urgency Manual), Wharewera-Mika et al^17^ highlighted the importance of considering particular sociocultural issues for indigenous people lest experience essential to those communities be overlooked and further marginalised. These measures do, however, also contain a potentially significant reminder of the shared nature of human experience and desire for personal growth that underpins much of our own experience and can be incorporated into clinical practice. The conclusion must be a reminder that clinical acts, including a focus on recovery, are essentially simultaneously personal and political and must be viewed through a critical and reflective lens to ensure that their purpose holds true to a practitioner's ethic of practice.

## Data Availability

Data availability is not applicable to this article as no new data were created or analysed in this study.
